# Whole Genome Sequence and Comparative Genomics of the Novel Lyme Borreliosis Causing Pathogen, *Borrelia mayonii*

**DOI:** 10.1371/journal.pone.0168994

**Published:** 2016-12-28

**Authors:** Luke C. Kingry, Dhwani Batra, Adam Replogle, Lori A. Rowe, Bobbi S. Pritt, Jeannine M. Petersen

**Affiliations:** 1 Division of Vector-Borne Diseases, Bacterial Diseases Branch, Centers for Disease Control and Prevention, Fort Collins, Colorado, United States of America; 2 Division of Scientific Resources, Biotechnology Core Facility Branch, Centers for Disease Control and Prevention, Atlanta, Georgia, United States of America; 3 Division of Clinical Microbiology, Department of Laboratory Medicine and Pathology, Mayo Clinic, Rochester, Minnesota, United States of America; University of North Dakota School of Medicine and Health Sciences, UNITED STATES

## Abstract

*Borrelia mayonii*, a *Borrelia burgdorferi sensu lato* (Bbsl) genospecies, was recently identified as a cause of Lyme borreliosis (LB) among patients from the upper midwestern United States. By microscopy and PCR, spirochete/genome loads in infected patients were estimated at 10^5^ to 10^6^ per milliliter of blood. Here, we present the full chromosome and plasmid sequences of two *B*. *mayonii* isolates, MN14-1420 and MN14-1539, cultured from blood of two of these patients. Whole genome sequencing and assembly was conducted using PacBio long read sequencing (Pacific Biosciences RSII instrument) followed by hierarchical genome-assembly process (HGAP). The *B*. *mayonii* genome is ~1.31 Mbp in size (26.9% average GC content) and is comprised of a linear chromosome, 8 linear and 7 circular plasmids. Consistent with its taxonomic designation as a new Bbsl genospecies, the *B*. *mayonii* linear chromosome shares only 93.83% average nucleotide identity with other genospecies. Both *B*. *mayonii* genomes contain plasmids similar to *B*. *burgdorferi sensu stricto* lp54, lp36, lp28-3, lp28-4, lp25, lp17, lp5, 5 cp32s, cp26, and cp9. The *vls* locus present on lp28-10 of *B*. *mayonii* MN14-1420 is remarkably long, being comprised of 24 silent *vls* cassettes. Genetic differences between the two *B*. *mayonii* genomes are limited and include 15 single nucleotide variations as well as 7 fewer silent *vls* cassettes and a lack of the lp5 plasmid in MN14-1539. Notably, 68 homologs to proteins present in *B*. *burgdorferi sensu stricto* appear to be lacking from the *B*. *mayonii* genomes. These include the complement inhibitor, CspZ (BB_H06), the fibronectin binding protein, BB_K32, as well as multiple lipoproteins and proteins of unknown function. This study shows the utility of long read sequencing for full genome assembly of Bbsl genomes, identifies putative genome regions of *B*. *mayonii* that may be linked to clinical manifestation or tissue tropism, and provides a valuable resource for pathogenicity, diagnostic and vaccine studies.

## Introduction

Members of the *Borrelia* genus group into two major disease causing clades, the Lyme borreliosis (LB) group and the relapsing fever (RF) group [1, 2]. Genospecies of the *Borrelia burgdorferi sensu lato* (Bbsl) complex are the causative agents of LB, the most common tick-borne disease in the northern hemisphere [3–6]. Humans acquire Bbsl infection by the bite of a hard tick, *Ixodes* spp., and in nature, small mammals or birds serve as hosts for Bbsl genospecies [4, 7, 8]. Although 20 Bbsl genospecies have been described worldwide, *B*. *afzelii*, *B*. *burgdorferi sensu stricto* (hereafter called *B*. *burgdorferi*), *B*. *bavariensis* and *B*. *garinii*, most commonly cause LB in Europe and Asia, whereas *B*. *burgdorferi* causes the majority of cases in North America [3]. Clinical features of LB are broad, variable, and disease manifestations seem to be linked with distinct tissue tropisms of specific Bbsl genospecies [9]. Early infection is typically localized and in the absence of treatment, the spirochete can disseminate to the nervous system (neuroborreliosis), joints (arthritis), heart (carditis), or other regions of the skin [10, 11]. *B*. *burgdorferi* is more often associated with Lyme arthritis as compared to other genospecies [9].

A novel Bbsl genospecies, *Borrelia mayonii*, was recently described as a cause of LB among six patients in the upper midwestern United States [12, 13]. Spirochetes isolated from two infected patients and taxonomically characterized using an eight gene multi-locus sequencing analysis (MLSA) demonstrated that the pairwise similarity to Bbsl genospecies fell below the system threshold for delineating Bbsl genospecies, confirming it as a novel genospecies [12]. For the six patients described, somewhat differing clinical presentations as compared to infection with *B*. *burgdorferi* were noted including, nausea or vomiting, symptoms potentially consistent with neurological effects, and a diffuse macular rash. Two of the six patients were hospitalized. By microscopy and quantitative PCR, *B*. *mayonii* infected patients were shown to have elevated numbers of spirochetes/genomes in their blood (10^5^ to 10^6^ spirochetes/genomes per milliliter) as compared to loads previously reported for *B*. *burgdorferi* (10^2^ to 10^3^ genomes per milliliter; 0.1 spirochete per milliliter) [14–16]. This elevated spirochetemia coupled with blood based PCR detection for five patients led to the suggestion that *B*. *mayonii* may have a distinct tissue tropism as compared to *B*. *burgdorferi* [11]. Patients’ infections were likely acquired by the bite of *I*. *scapularis*, which transmits *B*. *burgdorferi* in the United States. *I*. *scapularis* positive for *B*. *mayonii* were identified in several counties in Wisconsin and Minnesota [12, 13].

Bbsl genospecies possess one of the most unique and complex prokaryotic genomes [17, 18]. Their genomes are comprised of a long linear chromosome (~1Mb) and multiple (up to 21) linear and circular plasmids (collectively ~600 kbp), ranging in size from 5 to 56 kbp, of which several encode highly redundant sequence [17, 18]. The main chromosome of Bbsl genospecies is highly syntenic with low sequence variation across nearly the entire length [19]. This is in contrast to the extrachromosomal plasmids which are more structurally and genetically variable and encode proteins necessary for infection of vertebrate hosts and tick vectors, pathogenesis and immune cell invasion [19, 20]. The nomenclature developed for Bbsl plasmids is based on the reference strain *B*. *burgdorferi* B31. Plasmids from other Bbsl strains which show ≥95% percent identity to the PF32 plasmid partitioning proteins from B31 plasmids are named according to the B31 nomenclature [20]. The plasmids lp54 and cp26, have been observed in all characterized strains [21]. Species specific sequence differences in these two plasmids occur primarily in surface exposed genes [20]. Other plasmids are found in fewer than 10% of the analyzed Bbsl strains, while some plasmids are nearly always present but vary by size, gene content and copy number [21]. Cross species genomic comparisons suggest gene duplication/loss and differential expression patterns in addition to sequence variation in conserved lipoproteins are the primary drivers of differing tissue tropisms rather than gain of virulence genes [21–23].

Because of the similar sizes and highly paralogous nature of Bbsl plasmid sequences, next generation sequencing platforms have often yielded unfinished genome assemblies of Bbsl genospecies [22]. Short read platforms are typically unable to span the large number of repetitive regions present in *Borrelia* strains which in turn means the origin of the read cannot be precisely determined. PacBio^®^ sequencing offers the potential to overcome these limitations for Bbsl genospecies allowing for *de novo* assembly of full genomes. The long, multikilobase read lengths combined with high read coverage, can allow for accurate, non-hybrid, *de novo* assemblies that span problematic repetitive regions [24].

To investigate the genome structure of *B*. *mayonii* as well as genetic differences between *B*. *mayonii* and other Bbsl genospecies, we performed whole genome sequencing on two *B*. *mayonii* strains isolated from the blood of two acutely ill patients. Genomes were sequenced using the Pacific Biosciences RSII SMRT platform. By generating long reads (~6-15kb), with an average of >200X coverage, full genomes could be assembled and highly redundant plasmid sequences readily differentiated. These results demonstrate that the *B*. *mayonii* genome has a unique repertoire of plasmids and genetic features that distinguish it from other Bbsl genomes. Full chromosome and plasmid sequences for the *B*. *mayonii* MN14-1420 (type strain) and MN14-1539 genomes have been deposited in the NCBI database under BioProject # PRJNA321302.

## Materials and Methods

### Bacterial culture and DNA isolation

Low passage (P3) frozen stocks of two *B*. *mayonii* strains, MN14-1420 and MN14-1539, were grown in modified BSK at 34°C under microaerophilic conditions [13]. Spirochetes were pelleted by centrifugation at 8000 X g for 10 minutes. DNA was isolated from the pellet using the Qiagen DNA mini kit tissue protocol (Qiagen, Valencia, CA). Purified DNA was eluted in dH_2_0 and quantified using the Qubit fluorimeter (Life Technologies, Grand Island, NY).

### Library preparation and whole genome sequencing

Whole-genome sequencing was carried out using the PacBio RSII (Pacific Biosciences, Menlo Park, CA) platform. 5 μg of input genomic DNA was used for 10-kb fragment library preparation. DNA was sheared with g-TUBE^®^ microcentrifuge tubes (Covaris, Woburn, MA) and SMRTbell libraries prepared with DNA Template Kit 2.0 (Pacific Biosciences, Menlo Park, CA). Sequencing primers were annealed to the SMRTbell template and samples were sequenced using C3 chemistry, Polymerase version 5, SMRTAnalysis software version 2.3.0 and a single SMRT cell (Pacific Biosciences, Menlo Park, CA). Raw read quality and average reference consensus were determined using SMRTAnalysis software version 2.3.0.

### Genome assembly

*De novo* genome assembly of PacBio reads for MN14-1420 (49,701 reads totaling 343,091,509 bases, mean length 6,903 bp, max 34,633) and MN14-1539 (87,914 reads totaling 474,379,679 bases, mean length 5,395 bp, max 22,928) was conducted using the hierarchical genome-assembly process (HGAP3, SmrtAnalysis 2.3.0) with default parameters (expected genome size 2 Mbp, minimum target coverage 15X), which included consensus polishing with Quiver [24]. Briefly, raw reads were converted into high-quality pre-assembled reads which were assembled into contigs followed by a final consensus calling step to polish the assembled contigs. The mean quality score for the pre-assembled genomic reads was 85% for both strains. The assembly resulted in 15 and 14 polished contigs for MN14-1420 and MN15-1539 respectively. Each polished contig was quality assessed for topology by determining end to end overlap (circular) or long read-through of palindromic telomere sequence at the 5’ and 3’ ends of the contig (linear) using gepard 1.30 [25]. All contig assemblies were also subjected to re-sequencing analysis to validate trimmed regions and circular versus linear topology. Suspect circular contigs were trimmed to remove end to end overlap and re-oriented and stitched so that the circularized portion was located at the center of the contig. Reads were re-mapped to the trimmed re-oriented contig to ensure consistent coverage across the stitched (circularized) portion of the contig. Suspect linear contigs were trimmed to remove 5’ and 3’ sequence resulting from telomere read through. Reads were re-mapped to trimmed contigs to ensure consistent raw read coverage across the assembled genomes. The initial MN14-1539 assembly was missing two small contigs as compared to MN14-1420, a circular ~8 kbp contig and linear ~5 kbp contig. Reads from the MN14-1539 dataset were therefore mapped to the two contigs from MN14-1420. Reads corresponding to the circular ~8 kbp contig were present in MN14-1539 (92X coverage), while no trace reads mapped to the ~5 kbp linear contig. Sequence trimming and stitching was performed using CLC Genomics Workbench 8.0 (Qiagen, Valencia, CA). Re-sequencing analysis was conducted using SMRTAnalysis 2.3.0 (maxHits = 1, minAccuracy = 0.75, minLength = 50).

### Genome annotation

Genome annotation was performed using the NCBI Prokaryotic Genome Annotation Pipeline, to take advantage of the ability to combine annotation methods based on alignment with other Bbsl genospecies along with annotation methods predicting functional elements directly from sequence [26]. The annotation was compared between the two isolates to identify any major differences and overall percentages of gene specific ontology determined, i.e. hypothetical proteins, lipoproteins, pseudogenes, and plasmid partitioning genes. Additional lipoprotein prediction was performed using the spirochete specific (SpLip) algorithm with the *B*. *burgdorferi* prediction matrix [27].

Nomenclature for *B*. *mayonii* plasmids roughly followed the previously established criteria of naming plasmids according to the *B*. *burgdorferi* B31 nomenclature if PF32 plasmid partitioning proteins display ≥95% average amino acid identity (AAI) [20]. Additionally, if *B*. *mayonii* plasmids shared ≤95% AAI to B31 PF32 proteins, but ≥ 95% to PF32 proteins in other *B*. *burgdorferi* strains or genospecies, they were named thusly. For example, cp32-13 does not exist in B31, however the *B*. *mayonii* PF32 protein from cp32-13 shares 100% AAI to cp32-13 PF32 proteins in 3 *B*. *burgdorferi* strains (CA-11.2A, 72a, 118a). cp9 does not encode a PF32 protein, however the *B*. *mayonii* cp9 molecule contained all of the obvious genetic elements of cp9 from other Bbsl genospecies. The PF32 proteins from *B mayonii* lp28-4 and lp36 share 95% and 99% with lp-28-4 and lp36 PF32s from *B*. *bissettii* and *B*. *burgdorferi* strain 29805, respectively. *B*. *mayonii* lp28-10 was given a unique plasmid designation as its PF32 protein shares ≤ 89% AAI with PF32 proteins in the NCBI non-redundant protein database.

Chromosomal synteny was examined on a gene by gene basis by comparing the *B*. *burgdorferi* B31 chromosome and two *B*. *mayonii* chromosomes using a progressive Mauve 2.4.0 alignment [28]. The Mauve alignment was compared to the Core Genome comparison tool available at www.borreliabase.org [29]. Ideograms of each annotated plasmid were generated using CViT 1.2.1 (linear) and Circos 0.69 (circular) [30, 31]. Silent *vls* cassettes were determined using BLAST 2.2.30+ [32] by querying the lp28-10 plasmid DNA sequence with the DNA sequence from the predicted *vlsE* open reading frame (ORF) from the template strand at the 3’ terminus of the plasmid. Comparisons of silent *vls* cassette copy number between MN14-1420 and MN14-1539 were determined by whole plasmid alignments generated by STRETCHER [33]. Silent *vls* cassettes and PF54 array comparisons were visualized using EasyFig 2.1 [34].

### Comparative genomics

Two way average nucleotide identity (ANI) analysis was calculated using best hit and reciprocal best hit analysis (window size 1000 bp, step size 200 bp) as previously described (http://enve-omics.ce.gatech.edu/ani/) [35]. BLAST analyses between *B*. *mayonii* and *B*. *burgdorferi* B31 chromosomal and plasmid proteins were conducted using standalone BLAST 2.2.30+ and default blastp parameters. Chromosomal and plasmid proteins were compared separately. *B*. *burgdorferi* B31 homologs were identified using the “max_target_seqs 1” argument and an e-value ≥10^−2^. Each BLAST alignment with an e-value ≥10^−2^ was manually inspected. *B*. *burgdorferi* B31 plasmid proteins identified as absent were blasted against the NCBI non-redundant protein database to confirm they were present in at least one other *B*. *burgdoferi sensu stricto* strain and not unique to the *B*. *burgdorferi* B31 genome. Nucleotide sequence for *B*. *burgdorferi* genes considered missing from the *B*. *mayonii* genome were also queried against all unmapped reads from the two assemblies.

For comparison of proteins present on *B*. *mayonii* plasmids to other Bbsl genospecies, all individual protein sequence data was downloaded from NCBI (April 2016) for *B*. *burgdorferi* (n = 41,177), *B*. *garinii* (n = 17,334), *B*. *afzelii* (n = 11,502), *B*. *bissettii* (n = 2,946), and *B*. *miyamotoi* (n = 5,264). The downloaded proteins were converted to BLAST databases using the makeblastdb functionality in BLAST 2.2.30+. Each database was queried with a multi-fasta file comprised of each predicted protein from the individual *B*. *mayonii* plasmids and an average AAI determined for proteins greater than or equal to 100 amino acids in length. Each BLAST hit was inspected to ensure equivalent query coverage and alignment length. Phylogenetic analysis of a ~6.5 kbp region of cp26 from Bbsl genospecies was performed using MEGA6 [36]. Briefly, DNA sequences were aligned using MUSCLE, maximum likelihood trees generated using 4 discrete gamma categories and rooted at the midpoint based on Qiu et al 2014 [22], followed by 1000 bootstrap replicates. Sequences included in the phylogenetic analysis were retrieved from GenBank. Multi-alignment of the 26 amino acid C6 peptide was performed using Clustal Omega with default parameters [37].

### GenBank accession numbers

The two *B*. *mayonii* genome assemblies have been deposited in the GenBank public database under BioProject # PRJNA321302. BioSample, and individual Accession numbers for each DNA molecule are shown in Table 1.

**Table 1:** GenBank accession details for *B*. *mayonii* MN1420 and MN14-1539.

BioProject	BioSample	Strain	Plasmid name	Accession
PRJNA321302	SAMN04979181	MN14-1420	Chromosome	CP015780
PRJNA321302	SAMN04979181	MN14-1420	cp26	CP015781
PRJNA321302	SAMN04979181	MN14-1420	cp32-1	CP015782
PRJNA321302	SAMN04979181	MN14-1420	cp32-13	CP015783
PRJNA321302	SAMN04979181	MN14-1420	cp32-3	CP015784
PRJNA321302	SAMN04979181	MN14-1420	cp32-4	CP015785
PRJNA321302	SAMN04979181	MN14-1420	cp32-6	CP015786
PRJNA321302	SAMN04979181	MN14-1420	cp9	CP015787
PRJNA321302	SAMN04979181	MN14-1420	lp17	CP015788
PRJNA321302	SAMN04979181	MN14-1420	lp25	CP015789
PRJNA321302	SAMN04979181	MN14-1420	lp28-10	CP015790
PRJNA321302	SAMN04979181	MN14-1420	lp28-3	CP015791
PRJNA321302	SAMN04979181	MN14-1420	lp28-4	CP015792
PRJNA321302	SAMN04979181	MN14-1420	lp36	CP015793
PRJNA321302	SAMN04979181	MN14-1420	lp5	CP015794
PRJNA321302	SAMN04979181	MN14-1420	lp54	CP015795
PRJNA321302	SAMN04979182	MN14-1539	Chromosome	CP015796
PRJNA321302	SAMN04979182	MN14-1539	cp26	CP015797
PRJNA321302	SAMN04979182	MN14-1539	cp32-1	CP015798
PRJNA321302	SAMN04979182	MN14-1539	cp32-13	CP015799
PRJNA321302	SAMN04979182	MN14-1539	cp32-3	CP015800
PRJNA321302	SAMN04979182	MN14-1539	cp32-4	CP015801
PRJNA321302	SAMN04979182	MN14-1539	cp32-6	CP015802
PRJNA321302	SAMN04979182	MN14-1539	cp9	CP015962
PRJNA321302	SAMN04979182	MN14-1539	lp17	CP015803
PRJNA321302	SAMN04979182	MN14-1539	lp25	CP015804
PRJNA321302	SAMN04979182	MN14-1539	lp28-10	CP015805
PRJNA321302	SAMN04979182	MN14-1539	lp28-3	CP015806
PRJNA321302	SAMN04979182	MN14-1539	lp28-4	CP015807
PRJNA321302	SAMN04979182	MN14-1539	lp36	CP015808
PRJNA321302	SAMN04979182	MN14-1539	lp54	CP015809

## Results

### Genome sequencing and assembly of *B*. *mayonii*

**Fig 1:**
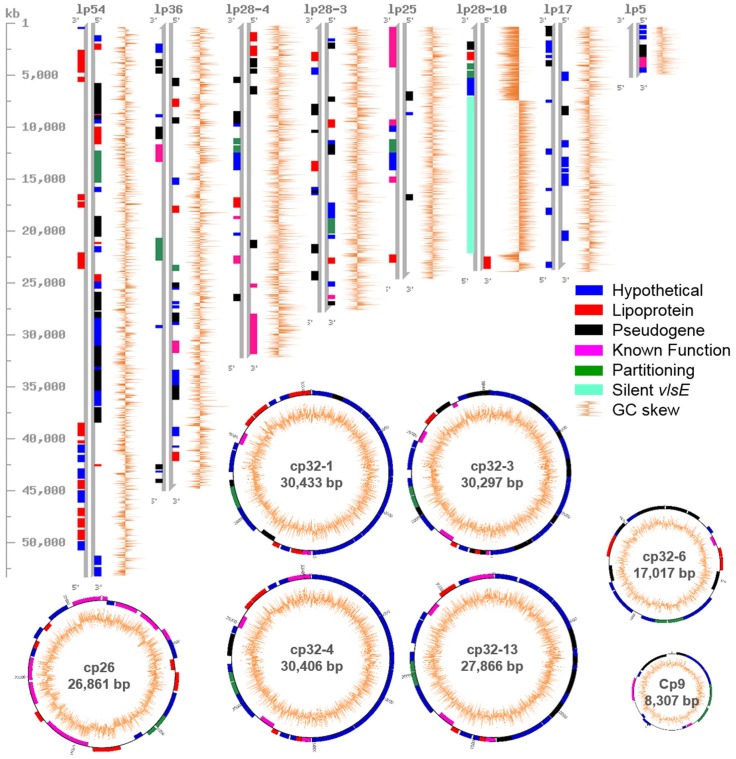
Full plasmid content of *B*. *mayonii*. Visual depiction and annotation of the linear and circular plasmids identified in *B*. *mayonii*. Linear plasmid (lp) ideograms were drawn with CViT 1.2.1 and are shown in relation to the size marker in kilobases (kb). Circular plasmid (cp) ideograms were drawn with Circos 0.69, circular plasmids are not drawn to scale. Annotation data was derived from the NCBI Prokaryotic Genome Annotation Pipeline. Proteins annotated as hypothetical (blue), lipoprotein (red), known function (pink), or plasmid partitioning (green) are indicated. Also shown are pseudogenes (black), the silent *vls* cassettes (light blue) and GC skew (orange lines), lp5 was only present in MN14-1420. lp28-10 is ~4 kbp shorter in MN14-1539.

For the two *B*. *mayonii* genomes, MN14-1420 and MN14-1539, the mean read length was 6149 bp and the average nucleotide coverage was 202X and 232X, respectively (range: 98X – 593X). The overall raw read quality was 0.85 with an average reference consensus of 99.99%. Complete contigs were generated for the chromosome and each of the extrachromosomal plasmids. The MN14-1420 genome is 1,311,545 bp in size (26.9% average GC content) and is comprised of a 904,387 bp linear chromosome, 8 linear plasmids and 7 circular plasmids (Table 2, Fig 1). It is predicted to encode 850 chromosomal genes and 411 plasmid-borne genes. The MN14-1539 genome is 1,293,788 bp and comprised of an identical sized linear chromosome, 7 linear and 7 circular plasmids. The MN14-1539 genome is predicted to encode 850 chromosomal genes and 405 plasmid-borne genes. Overall, the MN14-1420 and MN14-1539 genome sequences were highly similar and will therefore be discussed jointly, with detected differences noted. Size, average nucleotide coverage, GC content, and number of predicted open reading frames (ORF) is shown in Table 2 for the *B*. *mayonii* linear chromosome and each of the plasmids.

**Table 2:** Whole genome sequencing and annotation statistics for *B*. *mayonii* MN14-1420 and MN14-1539.

Contig	Size (bp)	Coverage (Avg)	Predicted Genes	Coding	rRNA	tRNA	GC%
Chromosome	904,387	208.4X	850	788	5	33	28.7
cp32-1	30,433	217.1X	42	39	0	0	29.3
cp32-3	30,297/30,362*	157.2X	43	31	0	0	28.7
cp32-4	30,406	160.4X	43	42	0	0	28.9
cp32-6	17,017	153.1X	22	15	0	0	27.2
cp32-13	27,866	174.8X	39	34	0	0	28.9
cp26	26,861/26,835*	145.5X	26	26	0	0	25.8
cp9	8,307	98.2X	8	6	0	0	24.2
lp54	53,359/53,343*	593.5X	62	45	0	0	27.0
lp36	44,035	218.8X	35	23	0	0	23.6
lp28-3	27,809	578.7X	27	18	0	0	24.2
lp28-4	32,294/32,269*	502.9X	20	13	0	0	24.2
lp28-10	23,800/19,566*	235.8X	8	7	0	0	39.4
lp25	24,702/24696*	139.1X	12	10	0	0	23.1
lp17	23,735/23,770*	403.9X	18	15	0	0	23.5
lp5	5,225**	309.7X	6	5	0	0	23.6

*Plasmid size differences displayed as MN14-1420/MN14-1539

**lp5 was not detected in the MN14-1539 genome

### *B*. *mayonii* chromosome

Sequence comparison of the linear chromosomes of MN14-1420 and MN14-1539 revealed an average nucleotide identity (ANI) of >99.99%, with no whole gene differences and only 9 single nucleotide polymorphisms (SNPs), two of which were non-synonymous (Table 3). The coding density of the *B*. *mayonii* chromosome is 95.2%. Analysis of the structural arrangement of the *B*. *mayonii* chromosome showed it is highly syntenic with chromosomes from other Bbsl genospecies (data not shown). When the sequence of the *B*. *mayonii* chromosome was compared with chromosomes available for 8 other Bbsl genospecies, the ANI was less than 94%. The highest ANI was to *B*. *burgdorferi* B31 at 93.83% followed by *B*. *bissetii* DN127 at 93.52% (S1 Table). The *B*. *mayonii* chromosome is predicted to contain 32 tRNAs, 5 rRNAs (16S, and two copies of 23 and 5S), 3 ncRNAs, 22 pseudogenes and 788 protein encoding genes. Whole chromosome BLAST analysis between *B*. *mayonii* and *B*. *burgdorferi* B31 revealed 763 (97%) proteins with greater than 80% amino acid identity (AAI), consistent with a highly conserved gene repertoire for the *B*. *mayonii* and *B*. *burgdorferi* chromosomes.

**Table 3:** Nucleotide differences detected between *B. mayonii* strains MN14-1420 and MN14-1539*.

Molecule	Position	Difference	MN14-1420	MN14-1539	Result
Chromosome	153,205	SNP	C	A	Synonymous
Chromosome	208,477	SNP	A	C	Non-synonymous
Chromosome	258,586	SNP	A	G	Synonymous
Chromosome	410,389	SNP	T	C	Synonymous
Chromosome	459,470	SNP	G	T	Intergenic
Chromosome	506,116	SNP	G	A	Synonymous
Chromosome	524,398	SNP	C	T	Synonymous
Chromosome	589,450	SNP	T	C	Synonymous
Chromosome	680,050	SNP	C	T	Non-synonymous
lp54	9,877	VNTR	CTAAACTTAATTAAAA	-	Intergenic
lp54	28456/28440**	SNP	A	G	Intergenic
lp54	33165/33149**	SNP	T	C	Synonymous
cp26	10,001	VNTR	TATGGAACTAACAGTTCCA	-	Intergenic
cp26	12204/12185**	VNTR	AAAACAT	-	Intergenic
lp25	24612	VNTR	TTAAAG	-	Intergenic
lp17	19980	VNTR	-	TAATTAATATATGATATAAATAAA	Intergenic
cp32-3	206	SNP	C	T	Synonymous
cp32-3	3926	SNP	T	G	Non-synonymous
cp32-3	22155	VNTR	-	CACTAAAATAGACAATGTTGAAAAGAATTTAAA	Intergenic
lp28-4	17602	VNTR	TAGTTCATCTTCTTCTTGTTTTTTCTT	-	Intergenic
lp28-4	23,645/23619**	INDEL	-	AT	Intergenic
lp28-3	3637	SNP	C	A	Non-synonymous
lp36	31962–3	SNP	AA	TT	Intergenic

*Excluding nucleotide differences in the *vls* locus of lp28-10

**Due to VNTR based differences in sequence length respective base position is displayed as MN14-1420/MN14-1539

SNP: Single Nucleotide Polymorphism, VNTR: Variable Number Tandem Repeat, INDEL: Insertion/Deletion

### Plasmid content of *B*. *mayonii*

Like other Bbsl genospecies, *B*. *mayonii* harbors a wide range of both linear and circular extrachromosomal plasmids totaling 407,158 bp, with 329 predicted protein coding genes and an average GC content of 26.7%. The overall coding density of the *B*. *mayonii* plasmids is 71.9%. Analogous Bbsl plasmids identified in *B*. *mayonii* include lp54, lp36, lp28-3, lp28-4, lp25, lp17, lp5, 5 cp32s, cp26, and cp9 (Fig 1). Sequence conservation and overall gene content and structural organization were noted for lp54, lp25, lp5, cp9, cp26, and the cp32s as compared to the respective plasmids in other Bbsl genospecies. The *B*. *mayonii* plasmids lp36, lp28-3, lp28-4, lp28-10, and lp17 show the greatest diversity in structural organization and gene content as compared to the respective plasmids from other Bbsl genospecies. One plasmid, lp5, is present in the MN14-1420 genome and absent from the MN14-1539 genome. Overall, the plasmid gene content of *B*. *mayonii* is characterized by a large number of predicted hypothetical proteins (59.3%), pseudogenes (19.9%), and lipoproteins (13.1%) (Fig 1). Only 6 SNPs, with the exception of the *vls* locus, were observed between the plasmid sequences of MN14-1420 and MN14-1539 (Table 3). Size differences noted between the cp32-3, cp26, lp54, lp28-4, lp25 and lp17 plasmids in MN14-1420 and MN14-1539 were due to either insertion/deletion (INDEL) or variable number of tandem repeats (VNTR), all of which were intergenic (Table 3).

**Fig 2:**
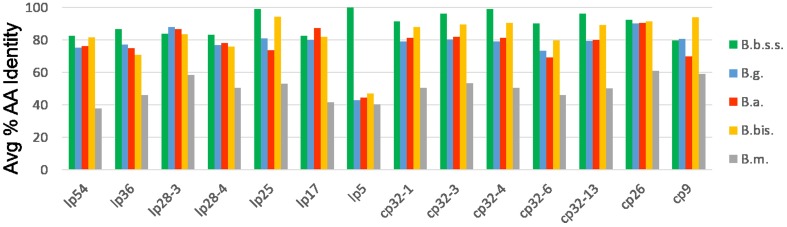
Plasmid based protein composition of *B*. *mayonii* in comparison to 4 other Bbsl genospecies and 1 RF *Borrelia* species. The mean amino acid identity is indicated for proteins present on each of the 14 *B*. *mayonii* plasmids. Comparisons are to protein databases constructed for *B*. *burgdorferi* (B.b.s.s.; green), *B*. *garinii* (B.g.; blue), *B*. *afzelii* (B. a.; red), *B*. *bissettii* (B.b.; yellow), and *B*. *miyamotoi* (B.m.; grey).

Amino acid sequences for all proteins encoded on the *B*. *mayonii* plasmids were compared to protein databases constructed for 4 Bbsl genospecies, *B*. *burgdorferi*, *B*. *bissettii*, *B*. *garinii* and *B*. *afzelii*, as well as the RF-like *Borrelia*, *B*. *miyamotoi*. Fig 2 shows the mean AAI of all proteins encoded on each of the 14 *B*. *mayonii* plasmids as compared to plasmid located proteins from the other *Borrelia* species. Lp28-10 was left out of the analysis as ORFs are predicted throughout the silent *vls* cassettes. Proteins present on 11 *B*. *mayonii* plasmids, lp54, lp36, lp28-4, lp25, lp5, cp32-1, cp32-3, cp32-4, cp32-6, cp32-13, and cp26, show the highest AAI at 82.6%, 86.6%, 83.2%, 99.3%, 100%, 91.7%, 96.3, 99.3%, 90.2%, 96.4%, and 92.6% respectively, to homologs present in *B*. *burgdorferi*.

Proteins present on three *B*. *mayonii* plasmids, lp28-3, lp17 and cp9, display slightly higher AAI to Bbsl genospecies other than *B*. *burgdorferi*. *B*. *mayonii* cp9 encoded proteins share the highest AAI (94.2%) to cp9 proteins from *B*. *bissettii*, whereas lp28-3 and lp17 had higher overall AAI to *B*. *garinii* and *B*. *afzelii* (88.2% and 87.5%, respectively). Each of the proteins on these two plasmids was further interrogated to identify those showing higher AAI to genospecies other than *B*. *burgdorferi*. The *B*. *mayonii* lp28-3 plasmid encodes a single hypothetical protein, Bmayo_04835 that displays 82 to 92% AAI identity to hypothetical proteins present in other genospecies as compared to only 50% AAI to a hypothetical protein present in the *B*. *burgdorferi* database. On lp17, a single predicted hypothetical protein, Bmayo_05225, returned poor amino acid similarity when compared to *B*. *burgdorferi*, *B*. *garinii*, *B*. *bissettii*, and *B*. *miyamotoi* proteins (0–80% AAI, 0–40% query coverage), whereas strong identity (89% AAI, 100% query coverage) to a hypothetical protein in *B*. *afzelii* was noted. When Bmayo_05225 was compared against the NCBI non-redundant protein database significant identity (88–89% AAI) was also detected to hypothetical protein homologs present in both *B*. *valaisiana* and *B*. *spielmanii*.

**Fig 3:**
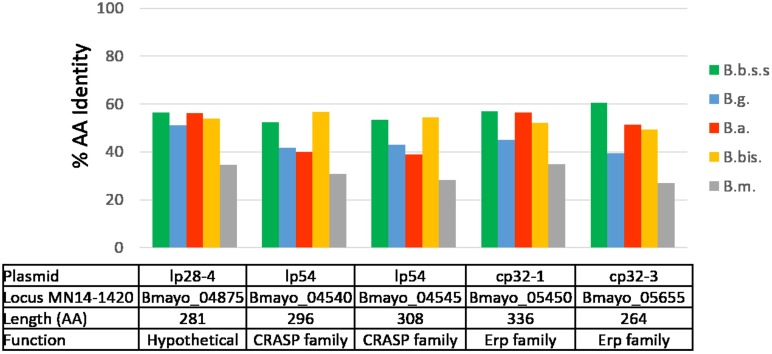
*B*. *mayonii* proteins with greatest sequence diversity as compared to 4 other Bbsl genospecies and 1 RF Borrelia species. Bar graph depicting *B*. *mayonii* proteins greater than 100 amino acids with less than 60% amino acid identity to other Bbsl proteins. Amino acid identity is shown with respect to homologs present in *B*. *burgdorferi* (B.b.s.s.; green), *B*. *garinii* (B.g.; blue), *B*. *afzelii* (B. a.; red), *B*. *bissettii* (B.b.; yellow) and *B*. *miyamotoi* (B.m.; grey).

No proteins unique to *B*. *mayonii* were identified and only five plasmid encoded proteins were detected that were ≥ 100 amino acids in length and shared ≤ 60% identity to proteins from other Bbsl species (Fig 3). Four of these proteins, Bmayo_04540, Bmayo_04545, Bmayo_05450, Bmayo_05655 are part of the CRASP (Complement Regulator-Acquiring Surface Protein) family, which are outer bacterial surface proteins involved in complement resistance, tissue tropism, and host cell binding of Bbsl genospecies (Reviewed in [38]). The two CRASP-1-like proteins (Bmayo_04540 and Bmayo_04545) encoded within the PF54 gene array of *B*. *mayonii* lp54 share from 39 to 56% AAI with CRASP proteins from other Bbsl genospecies. The two CRASP-3/4/5-like, also known as Erp family proteins (OspEF-related protein), encoded on cp32-1 and cp32-3 (Bmayo_05450 and Bmayo_05655), share from 39 to 60% AAI with CRASP-3/4/5 proteins from other Bbsl genospecies. The remaining protein encoded on lp28-4 is predicted as a hypothetical protein in *B*. *mayonii* and other Bbsl genospecies.

### *B*. *mayonii* cp26 and lp54

**Fig 4:**
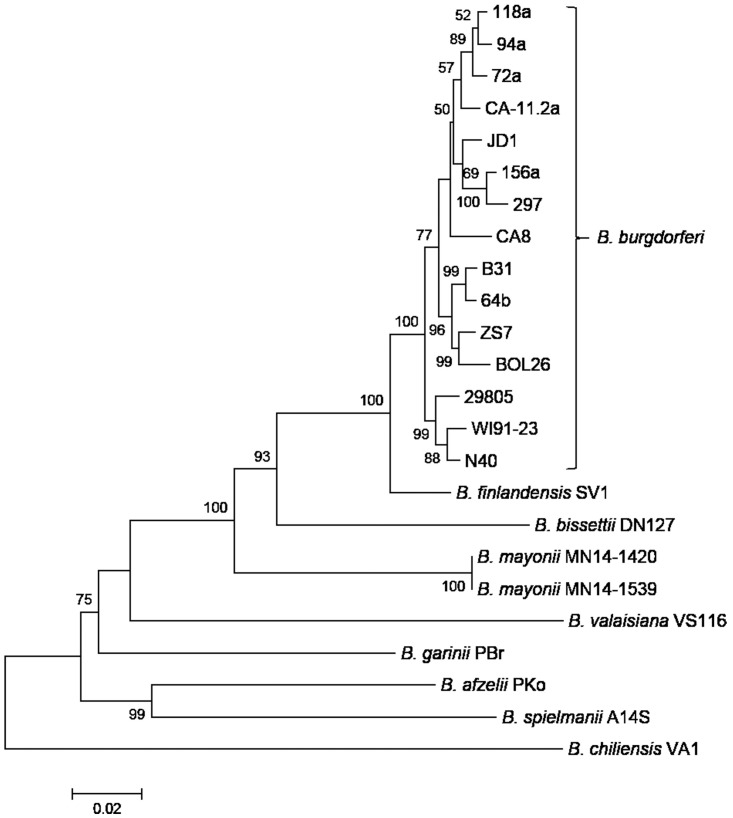
Phylogenetic analysis of *B*. *mayonii* based on a ~6.5 kbp region of cp26. The ~6.5 kbp region of cp26 encompasses open reading frames from BB_B14 through BB_B18 of *B*. *burgdorferi* B31. DNA sequences from 8 other Bbsl genospecies (22 strains) were retrieved from GenBank. Bootstrap values greater than 50% are shown. The scale bar corresponds to 0.02 substitutions per nucleotide position.

The conserved *B*. *mayonii* cp26 plasmid encodes a homolog of the OspC protein (Bmayo_06270). In *B*. *burgdorferi*, OspC is also encoded on cp26 and plays an important role in the invasion of tick salivary glands and mammalian infection [39, 40]. Between *B*. *mayonii* and *B*. *burgdorferi* B31, the OspC protein shares 76% AAI. The OspC proteins of *B*. *mayonii* MN14-1420 and MN14-1539 exhibit 100% AAI, which following the *B*. *burgdorferi* classification system, suggests both *B*. *mayonii* strains belong to the same allele group, termed here OspC type Bmayo1. Phylogenetic analysis of a ~6.5 kbp region of cp26, shown previously to be relatively free from recombination in Bbsl genospecies [22], is shown in Fig 4. The two *B*. *mayonii* strains form their own branch, distinct from *B*. *burgdorferi* and *B*. *bissettii*. In contrast to the *B*. *burgdorferi* strains, no genetic differences were detected across the 6.5 kbp region for the two *B*. *mayonii* strains. Within this ~6.5 kbp region, a 743 nucleotide stretch between genes Bmayo_06250 (hypothetical protein) and Bmayo_06255 (ABC transporter OppAIV) appears to be species specific for *B*. *mayonii*. BLAST analysis of the 743 nucleotide stretch against the NCBI non-redundant nucleotide database did not identify sequence similarity to any other *Borrelia* species.

**Fig 5:**
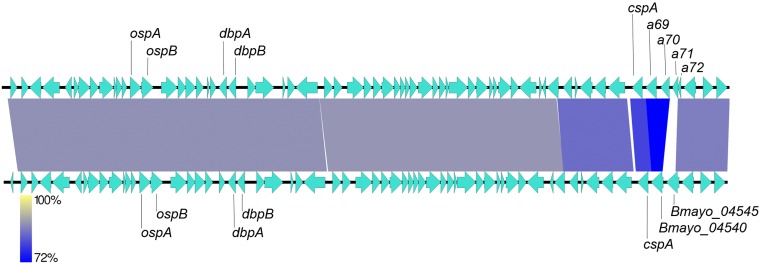
Comparison of *B*. *burgdorferi* B31 and *B*. *mayonii* lp54. Heat map of nucleotide identity between lp54 plasmids of *B*. *burgdorferi* B31 (top) and *B*. *mayonii* MN14-1420 (bottom). Nucleotide identity along the entire lp54 plasmids is displayed as colored blocks (Yellow = highest, Blue = lowest). Orientation of arrows indicates gene orientation on the forward or reverse DNA strand. The variable PF54 gene array is at the 3’ end of the plasmid. Specific gene loci discussed in text are indicated.

The lp54 plasmid is also present in *B*. *mayonii* and like other Bbsl lp54 plasmids, encodes the outer surface proteins, OspA and OspB (Bmayo_04320 and Bmayo_04325), which play an important role in colonization of tick vectors [41]. The OspA and OspB homologs in *B*. *mayonii* display 89% and 84% AAI to *B*. *burgdorferi* B31 OspA and OspB, respectively. Homologs to the lp54-borne decorin binding proteins, DbpA and DbpB (Bmayo_04355 and A7X69_04360), exhibit 67% and 73% AAI with *B*. *burgdorferi* B31, respectively. In addition to the aforementioned CRASP-1-like proteins (Bmayo_04540 and Bmayo_04545), a homolog of the CRASP CspA, (Bmayo_04535) is also encoded in the variable PF54 gene array and shares only 60% AAI to CspA from *B*. *burgdorferi* B31. The lower nucleotide identity between *B*. *burgdorferi* B31 and *B*. *mayonii* MN14-1420 across the PF54 gene array (3’ end of lp54) is highlighted in Fig 5. Nine proteins, BB_A05, 15, 25, 44, 52, 57, 65, 66, and 73, identified by Qiu *et al* [22] as highly variable among Bbsl genospecies and thus likely to be involved in host-pathogen interaction, were predicted as functional proteins in *B*. *mayonii*. Between *B*. *burgdorferi* B31 and *B*. *mayonii*, the AAI of these 9 proteins ranged from 67–90%.

### cp32 plasmids of *B*. *mayonii*

Multiple members of the cp32 plasmid family are present in all the analyzed Bbsl genospecies [42]. Despite the highly redundant sequence present among cp32 family members, maintenance in a single cell is possible due to unique plasmid partitioning genes present on each molecule that allow for discrete replication and division of the molecules amongst dividing cells [43, 44]. The *B*. *mayoniii* genome contains five different cp32s. The gene content and organization of the cp32-1, 3, 4, 6, and 13 plasmids in *B*. *mayonii* is highly conserved as compared to other Bbsl genospecies, with the exception of cp32-6, in which a truncation results in a 17 kbp circular plasmid and the loss of 21 ORFs as compared to other cp32’s. cp32-1, 3, 4, and 13 encode between 39 and 43 ORFs that share 74–100% AAI with proteins from the cp32 plasmids from other Bbsl species with the exception of the two Erp family proteins mentioned above. Each *B*. *mayonii* cp32 plasmid includes at least 1 Erp locus, while cp32-1 and cp32-4 each contain two 2 Erp loci.

### *B*. *mayonii* lp25 and lp5

*B*. *mayonii* lp25 and lp5 are the only other linear plasmids that share overall gene organization with plasmids from *B*. *burgdorferi*. *B*. *mayonii* lp25 encodes a homolog of the *B*. *burgdorferi* BptA (BB_E16) protein (Bmayo_05095) which has been shown to be associated with persistence of *B*. *burgdorferi* in the tick and is present on lp25 in other Bbsl genospecies [45]. The *B*. *mayonii* BptA homolog shares 94% AAI with *B*. *burgdorferi* B31 BptA. Homologs for both the nicotamidase (PncA, BB_E22) and restriction modification enzyme (BB_E02) are also encoded on *B*. *mayonii* lp25 and share 100% and 88% AAI with *B*. *burgdorferi* B31 BB_E22 and BB_E02, respectively. The small linear plasmid, lp5, was only present in *B*. *mayonii* MN14-1420. No sequence reads corresponding to this plasmid were detected in the MN14-1539 dataset, suggesting its absence from this strain. Previous studies have shown that plasmid stability varies in *B*. *burgdorferi*, with lp5 being lost at the highest frequency during laboratory passage [46]. The *B*. *mayonii* lp5 plasmid encodes five highly conserved proteins, four hypothetical (Bmayo_05230, Bmayo_05235, Bmayo_05240, Bmayo_05255) and one Sua5/YciO/YrdC/YwlC family protein (Bmayo_05250).

### *vls* containing lp28-10 in *B*. *mayonii*

**Fig 6:**
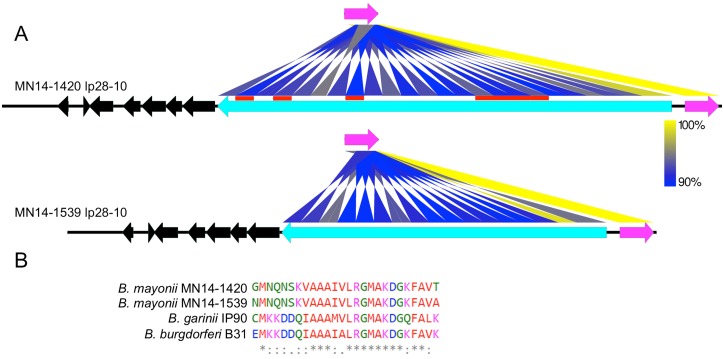
*vls* containing linear plasmid lp28-10 from *B*. *mayonii* MN14-1420 and MN14-1539. A) Heat map displaying nucleotide identity (blue—lowest; yellow—highest) between the active *vlsE* nucleotide sequence (magenta arrow) and silent *vls* cassettes within the two *B*. *mayonii* strains. Position and copy number of the silent cassette array is indicated by the blue to yellow ribbons. Red bars indicate silent *vls* loci present in MN14-1420 and missing in MN14-1539. Genes flanking the *vls* locus are indicated by black arrows. Orientation of arrows indicates gene orientation on the forward or reverse DNA strand. B) Multi-alignment of the 26 amino acid C6 peptide within VlsE from *B*. *mayonii*, *B*. *burgdorferi* B31 and *B*. *garinii* IP90. Identical (*), conserved (:) and differentially charged (.) amino acid residues are indicated.

A common feature amongst the lp28 plasmids of Bbsl genospecies is the *vls* locus, which encodes an antigentic variation system comprised of the active *vlsE* expression site (3’ terminus of the linear plasmid on the template strand) as well as multiple silent cassettes (*vls*), arranged head to tail on the complementary strand (Fig 6A). To evade the host immune response, the silent cassettes undergo rearrangements with the central region of the active *vlsE in vivo* in order to alter the protein sequence (reviewed in [47]). Comparison of the *vls* locus of the two *B*. *mayonii* strains revealed 24 silent *vls* cassettes in MN14-1420 as compared to 17 silent *vls* cassettes in MN14-1539 (Fig 6A). This variation in the number of silent *vls* cassettes results in a ~4kb size difference in the lp28-10 plasmids, which was independently confirmed by pulsed-field gel electrophoresis (data not shown). Alignment of the silent *vls* cassettes in MN14-1420 and MN14-1539 indicates that MN14-1539 is missing *vls*2/4/8 and *vls*15-18 (numbered left-to-right) as compared to MN14-1420, suggesting the loss or expansion of silent *vls* cassettes is not the result of a single recombination event (see red bars in Fig 6A). Of the 17 silent *vls* cassettes shared by MN14-1420 and MN14-1539, nucleotide identity ranged from 93% to 100%. The amino acid sequence of the VlsE protein of *B*. *mayonii* MN14-1420 and MN14-1539 shows 92% identity and only ~50% AAI with VlsE of B. *burgdorferi* B31. The highest AAI to VlsE in other Bbsl genospecies is to *B*. *finlandensis* SV1, at 77% AAI. The 26 amino acid region of VlsE, known as the C6 peptide, is conserved in *B*. *mayonii* with 14 identical amino acids and 10 conservative amino acid substitutions as compared to the *B*. *burgdorferi* B31 and *B*. *garinii* IP90 C6 peptide (Fig 6B).

### *B*. *burgdorferi* proteins lacking from *B*. *mayonii*

A notable feature of the *B*. *mayonii* genome is that it is comprised of fewer plasmids and is ~200 kbp smaller as compared to the *B*. *burgdorferi* B31 genome. Consistent with this, *B*. *burgdorferi* B31 plasmids encode 593 proteins, while *B*. *mayonii* plasmids encode only 329. The average plasmid encoded protein size is 222 amino acids for both *B*. *burgdorferi* B31 and *B*. *mayonii*. To investigate putative differences in the plasmid-borne protein composition of *B*. *burgdorferi* as compared to *B*. *mayonii*, sequences for the 593 *B*. *burgdorferi* B31 plasmid encoded proteins were compared to the predicted plasmid-borne proteome of *B*. *mayonii*. Sixty-eight plasmid encoded proteins in *B*. *burgdorferi* B31, which were present in at least one other *B*. *burgdorferi* strain, were also found to have an e-value greater than or equal to 10^−2^ when compared to *B*. *mayonii* plasmid encoded proteins (Table 4). In addition to e-value, percent identity, bit score, and query coverage were also evaluated (Table 4). Many of these proteins (52%) are located on plasmids in *B*. *burgdorferi* B31 for which there is no equivalent (e.g. lp28-2, lp38) in the *B*. *mayonii* genome. Fifty-five of the 68 proteins lacking from *B*. *mayonii* are predicted as hypothetical proteins (81%) with unknown function, 8 as lipoproteins (11%), and only 7 (10%) assigned a known function. Most interestingly, a homolog of the fibronectin binding protein (BB_K32) is not present on *B*. *mayonii* lp36 or elsewhere in the genome. Additionally, a homolog of the CRASP family protein, CspZ (BB_H06) is lacking in *B*. *mayonii*.

**Table 4:** Putative *B*. *burgdorferi* plasmid-borne proteins absent from the *B*. *mayonii* genome.

Gene*	Protein	% ID	E-Value	Bit Score	Query Coverage (%)	Accession #*	Size
BB_A58	hypothetical protein	40	0.05	24.3	37	NP_045731.1	67
BB_D001	lipoprotein	50	0.062	23.9	28	YP_004940415.1	64
BB_D0031	hypothetical protein	36.36	0.4	20.8	60	YP_004940417.1	53
BB_D01	hypothetical protein	31.25	2.3	21.6	20	NP_045385.1	157
BB_D24	hypothetical protein	33.33	6.9	18.1	34	NP_045406.1	76
BB_F0039	hypothetical protein	41.67	3.4	18.5	23	YP_004940412.1	52
BB_F02	hypothetical protein	50	3.3	20.4	15	NP_045438.1	118
BB_F08	hypothetical protein	40.63	1.6	19.6	58	NP_045444.1	55
BB_F17	putative transmembrane protein	32.56	3.7	19.2	51	YP_004940411.1	85
BB_G0036	hypothetical protein	44.44	0.26	23.5	23	YP_004940633.1	112
BB_G02	hypothetical protein	41.94	0.05	27.7	11	NP_045464.1	293
BB_G12	hypothetical protein	28.24	0.13	25	51	NP_045472.1	142
BB_G13	hypothetical protein	31.43	0.53	24.6	13	NP_045473.2	272
BB_G14	hypothetical protein	50	1.4	21.9	14	NP_045474.1	147
BB_G15	hypothetical protein	30.91	0.52	23.1	42	NP_045475.2	130
BB_G16	hypothetical protein	29.55	0.011	27.3	39	NP_045476.1	112
BB_G17	hypothetical protein	22.12	4.7	21.6	38	NP_045477.1	277
BB_G18	hypothetical protein	36.84	2.5	21.2	11	NP_045478.1	169
BB_G19	hypothetical protein	23.38	0.21	25	42	NP_045479.1	181
BB_G20	hypothetical protein	23.58	0.37	25.4	29	NP_045480.1	341
BB_G21	hypothetical protein	40.74	2.5	23.1	7	NP_045481.1	396
BB_G22	hypothetical protein	38.3	1.2	23.5	17	NP_045482.1	265
BB_G23	hypothetical protein	40	2.3	22.3	12	NP_045483.1	260
BB_G24	hypothetical protein	23.64	5.8	23.1	6	NP_045484.2	879
BB_G26	hypothetical protein	25.68	0.87	22.3	52	NP_045486.1	124
BB_G28	hypothetical protein	36.59	0.36	23.5	32	NP_045488.1	128
BB_G30	hypothetical protein	28.38	2.1	21.2	54	NP_045490.2	136
BB_G32	replicative DNA helicase	40.91	0.36	24.6	8	NP_045492.1	275
BB_G34	hypothetical protein	24.32	1.2	23.1	29	NP_045494.1	254
BB_H02	hypothetical protein	23.13	1.8	21.9	73	NP_045496.1	155
BB_H06	complement regulator-acquiring surface protein (CspZ) (lipoprotein)	33.33	1.3	23.1	15	NP_045500.1	236
BB_H09a	hypothetical protein	40.63	1.2	21.9	23	YP_004940634.1	137
BB_H17	hypothetical protein	35	5.4	17.7	52	NP_045510.1	63
BB_H25	hypothetical protein	33.33	4	19.2	46	NP_045516.1	81
BB_H40	transposase-like protein	31.37	1	22.3	33	NP_045530.1	155
BB_I18	hypothetical protein	27.27	1.3	19.6	44	NP_045549.1	50
BB_I41	hypothetical protein	32.69	1.8	20.4	56	NP_045572.1	80
BB_J0056	hypothetical protein	40.54	1	20	70	YP_004940639.1	53
BB_J0058	hypothetical protein	37.5	0.12	22.3	47	YP_004940640.1	51
BB_J11	hypothetical protein	31.67	0.31	21.6	95	NP_045635.1	61
BB_J13	hypothetical protein	28.57	0.52	21.9	53	NP_045637.1	92
BB_J23	hypothetical protein	28.3	0.23	25	19	NP_045647.2	269
BB_J25	hypothetical protein	28.89	0.54	24.6	13	NP_045649.2	346
BB_J27	efflux ABC transporter permease	32.31	0.79	24.6	15	NP_045651.2	409
BB_J28	hypothetical protein	24.69	0.083	26.6	61	NP_045652.2	241
BB_J29	hypothetical protein	50	0.11	25	7	NP_045653.2	321
BB_J31	hypothetical protein	17.74	0.67	23.9	26	NP_045655.1	240
BB_J36	lipoprotein	37.21	0.73	24.3	12	NP_045660.1	352
BB_J37	hypothetical protein	23.26	4.5	18.1	84	NP_045661.2	51
BB_J43	hypothetical protein	50	0.1	25	7	NP_045667.1	306
BB_J45	lipoprotein	17.74	0.55	24.3	26	NP_045669.1	240
BB_J46	hypothetical protein	29.63	0.7	21.9	49	NP_045670.1	98
BB_J48	hypothetical protein	27.17	0.12	25.8	86	NP_045672.2	204
BB_K0058	hypothetical protein	22	2.3	19.2	78	YP_004940637.1	58
BB_K0059	hypothetical protein	38.1	0.17	22.3	34	YP_004940638.1	61
BB_K07	lipoprotein	29.25	0.25	25.4	40	NP_045581.1	250
BB_K09	hypothetical protein	43.48	0.63	21.9	24	NP_045583.1	94
BB_K32	fibronectin-binding protein (lipoprotein)	43.48	0.07	27.7	6	NP_045605.1	354
BB_K34	hypothetical protein	53.33	2	20	21	NP_045607.1	73
BB_K35	hypothetical protein	40	5.5	17.7	26	NP_045608.1	57
BB_K41	hypothetical protein	27.78	1.1	22.7	35	NP_045613.2	181
BB_K42	hypothetical protein	58.82	0.15	22.7	24	NP_045614.1	72
BB_K47	hypothetical protein	30	0.069	27.7	18	NP_045618.1	328
BB_K48	immunogenic protein P37 (lipoprotein)	47.83	0.066	27.3	8	NP_045619.1	288
BB_M39	hypothetical protein	26.83	1.8	21.9	28	NP_051330.1	222
BB_Q62	hypothetical protein	27.5	0.15	24.3	32	NP_051521.1	124
BB_Q88	hypothetical protein	31.25	2.2	21.6	20	NP_051536.1	157
BB_Q89	lipoprotein	50	0.062	23.9	28	NP_051537.1	64

*Gene name and accession numbers are derived from the *B*. *burgdorferi* B31 genome.

## Discussion

The full genomes, including chromosome and plasmid sequences, for two *B*. *mayonii* strains (MN14-1420 and MN14-1539) cultured from the blood of two patients are presented in this report. The recently discovered cause of LB in the United States, *B*. *mayonii* has thus far only been detected in patients and ticks from the upper midwest [12, 13]. Overall, the genomes of the two strains are very similar, being comprised of a 904,387 bp linear chromosome with only 9 SNP differences. The MN14-1420 genome includes 407,158 bp of extrachromosomal plasmid (235,971 bp linear, 171,187 bp circular) DNA while the MN14-1539 genome contains 397,714 bp of extrachromosomal plasmid DNA (226,488 bp linear, 171,226 bp circular), with the primary difference due to a 4 kbp size difference in the *vls* containing plasmid, lp28-10, and the absence of the 5 kbp lp5 plasmid in MN14-1539. It’s likely the lp5 plasmid was lost from MN14-1539 during cultivation of the spirochete *in vitro*, as the corresponding plasmid in *B*. *burgdorferi* has been shown to be lost at the highest frequency during laboratory passage [46]. Notably, between the ~400,000 bp of plasmid sequence shared by MN14-1420 and MN14-1539 (excluding the *vls* locus), only 6 SNPs, 1 INDEL and 7 VNTRs were detected. As the two isolates were recovered from patients exposed in different geographic regions of the upper midwest, these findings suggest that *B*. *mayonii* strain diversity in this region may be limited [12, 13].

The genome sequence of *B*. *mayonii* confirms previous taxonomic findings from 8 housekeeping gene MLSA identifying it as a novel genospecies of Bbsl [12, 13]. Here, nucleotide comparison of the entire linear chromosome of *B*. *mayonii* with other Bbsl genospecies revealed an ANI ≤93.8%. An ANI value of 95% corresponds with a DNA-DNA hybridization value of 70%, the threshold historically used for bacterial species classification [35]. An additional, plasmid based phylogeny inferred from a 6.5 kbp segment of cp26 similarly demonstrates *B*. *mayonii* forms a clade distinct from other known Bbsl genospecies. Lastly, the *B*. *mayonii* linear plasmids lp28-3, lp28-4, lp28-10, lp36 and lp17 all possess unique gene order and gene content compared to similar plasmids in *B*. *burgdorferi* B31. For example, *B*. *mayonii* lp36 lacks 11 other proteins normally present on lp36. Likewise, the first 13 ORFs present on *B*. *burgdorferi* B31 lp17 are either missing from *B*. *mayonii* lp17 or present on alternative plasmids.

Features of the *B*. *mayonii* genome in common with other Bbsl genomes include a conserved, syntenic chromosome with high coding density (>95%), several linear and circular plasmids with significantly lower coding density (<72%), a high number of pseudogenes, and a large number of putative proteins of unknown function. Plasmids similar to lp54, lp25, lp5, cp26 and cp32 in other Bbsl genospecies, including largely conserved gene orders, were identified in *B*. *mayonii*. Of these plasmids, the lowest AAI to other Bbsl genospecies was for lp54. This is primarily due to the sequence diversity in the 3’ PF54 gene array which includes CspA (Bmayo_04535) and the two CRASP-1-like proteins, Bmayo_04540 and Bmayo_04545. Significant sequence divergence of the PF54 gene array between different Bbsl genospecies has been previously described and postulated to reflect adaptation to different host species [22].

During mammalian infection, the *vlsE* expression site of LB spirochetes undergoes extensive sequence recombination with the silent *vls* cassettes in order to evade host immune defenses [48, 49]. Despite overall amino acid diversity among VlsE proteins of various Bbsl genospecies, the C6 peptide is conserved and thus widely used as a serologic diagnostic marker for detection of antibodies produced by infected patients [50, 51]. Here, sequence analysis confirmed that the VlsE C6 peptide sequence in *B*. *mayonii* is also conserved. Consistent with this, serum samples collected ≥3 days of illness onset from *B*. *mayonii* infected patients were seropositive when tested using a C6 *B*. *burgdorferi* (Lyme) ELISA assay [11]. Across the entire *vls* locus, only limited comparisons with other Bbsl genospecies species was possible due to the small number of complete assemblies publicly available, two from *B*. *burgdorferi*, strains B31 and JD1, and one from *B garinii*, strain Far04 [20, 47, 52]. The two *B*. *burgdorferi* strains contain 15 and 14 silent *vls* cassettes while the *B*. *garinii* strain includes 19 silent *vls* cassettes. In comparison, the silent *vls* cassette of *B*. *mayonii* strains, MN14-1420 and MN14-1539, is comprised of 24 and 17 silent copies, respectively. Considering the small amount of nucleotide variation detected between the two *B*. *mayonii* strains, the observed differences in silent *vls* cassette copy number and nucleotide identity was notable. Moreover, the location of silent *vls* cassettes present in MN14-1420 as compared to MN14-1539 indicates that silent cassette expansion or reduction most likely occurs throughout the cassette rather than only at the 5’ or 3’ ends. The direct repeats bookending each of the silent *vls* cassettes typically make assembly of this region challenging, especially if sequencing platforms yielding short read lengths are utilized. Within the ~200X sequence read coverage of the *B*. *mayonii* lp28-10 plasmids, multiple overlapping reads greater than 10kbp spanned the entire *vls* locus as well as flanking regions which allowed for complete assembly. Thus, long read genome sequencing provides a new tool for studying the complete *vls* locus of Bbsl genospecies in greater detail.

Given the reported spirochetemia levels in *B mayonii* infected patients and detection of the organism in *I*. *scapularis* ticks, proteins encoded in the *B*. *mayonii* genome were analyzed for similarity to those present in the *I*. *scapularis* transmitted relapsing fever-like spirochete, *B*. *miyamotoi*. No proteins with significantly higher identity to proteins in *B*. *miyam*otoi as compared to other Bbsl genospecies were detected. The ability of relapsing fever *Borrelia* to grow to very high numbers in the blood of infected patients differs from LB *Borrelia* and has been attributed, in part, to an intact purine salvage pathway (*hpt*, *purA* and *purB*) and complete purine metabolism pathway (*nrdIEF*) [53]. As observed for other Bbsl genospecies, homologs of the proteins encoded in the *nrdIEF/purAB* operons of relapsing fever borreliae were also not present in the *B*. *mayonii* genome.

The genetic basis for differences among Bbsl genospecies with respect to tissue tropism and/or clinical manifestations is not well understood. Outer surface lipoproteins important for tissue colonization (DbpA, BBK_32) and evasion of complement-dependent killing (CRASPs) have been linked to tropism and/or pathogenic differences [38, 54–57]. In the *B*. *mayonii* genome, the proteins displaying the highest sequence variation as compared to other Bbsl genospecies include DpbA (Bmayo_04355) as well as five CRASP family proteins [Bmayo_04530 (CspA), Bmayo_04535, Bmayo_04540, Bmayo_04545, Bmayo_05450, Bmayo_05655], three of which are encoded within the PF54 gene array. Additionally, our analyses indicate that the *B*. *mayonii* genome lacks homologs to two proteins involved in tissue colonization and immune evasion by *B*. *burgdorferi*: the fibronectin binding protein, BB_K32 [57–59] and CspZ, a complement inhibitor with no known paralogs [60, 61]. Whether these differences might be linked to a distinct tissue tropism or pathogenicity of *B*. *mayonii* requires further characterization. Description of illness in additional patients infected with *B*. *mayonii* will also be important for increasing our understanding of the breadth of clinical manifestations.

Another notable feature of the *B*. *mayonii* genome is that at least 68 plasmid-borne proteins present in *B*. *burgdorferi*, including BBK_32 and CspZ, appear to be lacking. Interestingly, most of these proteins (52%) are located on plasmids in *B*. *burgdorferi* B31 for which there is no equivalent (e.g. lp28-2, lp38) in the *B*. *mayonii* genome. Of the 68 proteins, all are present in *B*. *burgdorferi* strains other than B31, indicating the identified proteins lacking from *B*. *mayonii* are not *B*. *burgdorferi* B31 strain specific. Given, the paralogous nature of *Borrelia* genomes, we cannot rule out the possibility that functional domains of these *B*. *burgdorferi* proteins may be present elsewhere in *B*. *mayonii*. Additionally, it’s possible other *B*. *burgdorferi* proteins may be lacking from the *B*. *mayonii* genome and were not detected here due to the stringency of the comparisons performed.

Currently, relatively few complete genome assemblies are available for Bbsl genospecies, despite being the causative agents of the most commonly reported tick-borne infection in the northern hemisphere. We have shown here the feasibility of using long read sequencing technology, specifically PacBio, coupled with high density sequence coverage, for non-hybrid, *de novo* assembly of complete Bbsl genomes. Future utilization of long read sequencing to generate complete genome assemblies should enable a more comprehensive picture of genetic diversity, gene content differences, and structural organization of Bbsl plasmid sequences. Sequencing of additional *B*. *mayonii* strains from both environmental and human samples will be important for understanding genetic diversity of this species as well as for confirming the detected differences between *B*. *burgdorferi* and *B*. *mayonii*. The genome sequences presented here should provide a valuable resource for future work to characterize the pathobiology and ecology of *B*. *mayonii*, and a foundation for diagnostic and vaccine work.

## Supporting Information

S1 Table Average nucleotide identity of the *B*. *mayonii* linear chromosomes as compared to 8 other Bbsl genospecies. ANI was calculated using 2-way reciprocal best hits between the chromosomes of *B*. *mayonii*, *B*. *burgdorferi* B31, *B*. *garinii* PBR, *B*. *bavariensis* PBi, *B*. *afzelii* PKo, *B*. *bissettii* DN127, *B*. *finlandensis* SV1, *B*. *spielmanii* A14S, *B*. *valaisiana* VS116.
(DOCX)
