# Supplementary material for: Whole Genome Sequence and Comparative Genomics of the Novel Lyme Borreliosis Causing Pathogen, Borrelia mayonii
Source: PLoS One. 2016 Dec 28;11(12):e0168994. doi: 10.1371/journal.pone.0168994 (PMC5193363; doi:10.1371/journal.pone.0168994)
Supplement: S1 Table — ANI was calculated using 2-way reciprocal best hits between the chromosomes of B. mayonii, B. burgdorferi B31, B. garinii PBR, B. bavariensis PBi, B. afzelii PKo, B. bissettii DN127, B. finlandensis SV1, B. spielmanii A14S, B. valaisiana VS116. (DOCX) [file pone.0168994.s001.docx]

| Species/Strain | MN14-1539 | MN14-1420 | B31 | PBr | PBi | PKo | DN127 | SV1 | A14S |
| --- | --- | --- | --- | --- | --- | --- | --- | --- | --- |
| *B. mayonii* MN14-1539 |  |  |  |  |  |  |  |  |  |
| *B. mayonii* MN14-1420 | 99.99 |  |  |  |  |  |  |  |  |
| *B. burgdorferi* B31 | 93.83 | 93.83 |  |  |  |  |  |  |  |
| *B. garinii* PBr | 91.79 | 91.78 | 91.39 |  |  |  |  |  |  |
| *B. bavariensis* PBi | 91.79 | 91.8 | 91.35 | 97.35 |  |  |  |  |  |
| *B. afzelii* PKo | 91.94 | 91.92 | 91.52 | 92.38 | 92.46 |  |  |  |  |
| *B. bissettii* DN127 | 93.52 | 93.51 | 94.06 | 91.1 | 91.03 | 91.21 |  |  |  |
| *B. finlandensis* SV1 | 93.73 | 93.74 | 97.93 | 91.41 | 91.39 | 91.52 | 93.91 |  |  |
| *B. spielmanii* A14S | 91.23 | 91.23 | 90.83 | 91.66 | 91.71 | 93.72 | 90.59 | 90.86 |  |
| *B. valaisiana* VS116 | 91.66 | 91.65 | 91.3 | 92.06 | 92.05 | 92.17 | 90.98 | 91.26 | 91.49 |

**S1 Table. Average nucleotide identity of the *B. mayonii* linear chromosomes as compared to 8 other Bbsl genospecies.**
